# Supplementary material for: Benefits of Range-Separated Hybrid and Double-Hybrid Functionals for a Large and Diverse Data Set of Reaction Energies and Barrier Heights
Source: J Phys Chem A. 2022 Aug 5;126(32):5492–505. doi: 10.1021/acs.jpca.2c03922 (PMC9393870; doi:10.1021/acs.jpca.2c03922)
Supplement: Supplementary file 1 — jp2c03922_si_001.pdf [file jp2c03922_si_001.pdf]

Supporting Information for:

Benefits of Range-Separated Hybrid and Double-Hybrid Functionals for a Large and Diverse Data Set of Reaction Energies and Barrier Heights

Golokesh Santra, Rivka Calinsky, and Jan M.L. Martin*

Department of Molecular Chemistry and Materials Science, Weizmann Institute of Science, 7610001 Reḥovot, Israel.

Email: gershom@weizmann.ac.il

Table S1: Final parameters for our ωDSD-PBEP86-D3BJ, ωDOD-PBEP86-D3BJ and ωnoDispSD-PBEP86 functionals with different fraction of HF exchange and range separation parameter (ω).

**Functionals**	**Parameters[1]**					
	**x/100 or $c_{X,HF}$**	**ω**	**c_{DFT}**	**c_{2ab}**	**c_{2ss}**	**s_6**
**ωDSDx-PBEP86-D3BJ**	0.20	0.30	0.5858	0.1579	0.0958	0.9053
	0.40	0.30	0.4878	0.3589	0.1579	0.6580
	0.50	0.30	0.4400	0.4672	0.1839	0.5263
	0.60	0.22	0.4130	0.5477	0.1386	0.4933
		0.30	0.3912	0.5796	0.2075	0.4028
	0.69	0.10	0.3881	0.6275	0.1004	0.4088
		0.16	0.3659	0.6459	0.1494	0.3857
		0.20	0.3576	0.6551	0.1767	0.3662
		0.25	0.3514	0.6677	0.2049	0.3398
		0.30	0.3379	0.6877	0.2481	0.2846
	0.72	0.08	0.3674	0.6638	0.1200	0.3561
		0.13	0.3531	0.6738	0.1537	0.3476
**ωDODx-PBEP86-D3BJ**	0.20	0.30	0.6091	0.2052	0	0.9278
	0.40	0.30	0.5247	0.4211	0	0.7131
	0.50	0.30	0.4751	0.5362	0	0.6075
	0.60	0.22	0.4403	0.5976	0	0.5542
		0.30	0.4248	0.6686	0	0.5005
	0.69	0.10	0.4063	0.6611	0	0.4683
		0.16	0.4003	0.6920	0	0.4561
		0.20	0.3905	0.7216	0	0.4509
		0.25	0.3961	0.7477	0	0.4216
		0.30	0.3869	0.7838	0	0.3936
	0.72	0.08	0.3927	0.7025	0	0.4235
		0.13	0.3840	0.7285	0	0.4202
**ωnoDispSDx-PBEP86**	0.20	0.30	0.6098	0.1261	0.5507	0
	0.40	0.30	0.5055	0.3286	0.4997	0
	0.50	0.30	0.4529	0.4350	0.4733	0
	0.60	0.30	0.3799	0.5675	0.4512	0
		0.22	0.4034	0.5137	0.4476	0
	0.69	0.10	0.3762	0.5747	0.3922	0
		0.16	0.3578	0.5997	0.4147	0
		0.20	0.3474	0.6291	0.4142	0
		0.25	0.3423	0.6489	0.4131	0
		0.30	0.3224	0.6774	0.4277	0
	0.72	0.08	0.3520	0.6185	0.3848	0
		0.13	0.3385	0.6384	0.3952	0

[1] s_8=0, a_1=0, and a_2=5.6 throughout.

Table S2: Root-mean-square deviations (RMSDs, kcal/mol) for different DFT functionals on the complete BH9 barrier height set and its nine subsets. The subsets (or the reaction types) of the BH9 are radical rearrangement (I), Diels-Alder (II), halogen atom transfer (III), hydrogen atom transfer (IV), hydride transfer (V), Boron and Silicon containing reactions (VI), proton transfer (VII), nucleophilic substitution (VIII), and nucleophilic addition (IX). Heatmapping is from red (worst) via yellow to green (best).

	RMSD (kcal/mol)									
**Functionals**	**I**	**II**	**III**	**IV**	**V**	**VI**	**VII**	**VIII**	**IX**	**Total**
B97M-V	2.89	6.31	8.61	7.48	10.84	3.40	2.26	4.30	3.27	6.70
BMK-D3BJ	2.19	2.94	2.84	5.29	3.95	3.88	1.13	1.68	1.79	3.54
BMK	1.79	3.99	2.43	2.72	5.64	2.88	2.35	4.47	1.74	3.45
ωB97X-D	1.13	2.15	1.96	3.76	1.99	2.28	1.04	2.61	1.13	2.42
ωB97X-V	1.60	4.26	1.32	2.70	3.63	2.25	0.99	3.09	1.90	3.12
ωB97M-V	0.98	1.61	1.67	2.53	3.47	1.73	0.76	1.92	1.03	2.01
ωB97X-2-D3BJ	2.24	4.33	2.19	3.48	4.91	1.57	1.55	2.09	1.82	3.48
ωB97X-2	2.24	4.33	2.19	3.48	4.90	1.57	1.55	2.08	1.82	3.48
ωB97M(2)	1.15	1.38	2.01	3.03	1.42	1.24	0.67	1.08	0.91	1.83
PBE-D3BJ	5.57	10.93	13.78	12.89	17.20	7.00	6.85	8.38	7.00	11.39
PBE	4.92	9.62	11.33	9.67	10.99	4.78	6.19	5.21	5.74	8.90
PBE20	2.26	4.36	4.92	5.33	4.75	2.53	3.64	2.04	2.86	4.24
LRC-ωPBEh	1.84	3.34	2.95	3.07	5.28	2.84	2.53	5.37	1.47	3.32
PBE0-2-D3BJ	4.53	4.91	2.89	3.61	7.29	2.73	1.98	1.26	2.33	4.38
PBE0-2	4.56	4.29	3.29	3.02	5.48	2.52	1.73	1.71	1.96	3.81
SOS0-PBE0-2-D3BJ	4.99	2.63	5.02	2.43	2.21	2.04	0.71	1.91	1.33	3.07
SOS0-PBE0-2	5.27	2.94	6.30	3.36	2.35	3.43	1.11	3.96	1.33	3.72
PBE0-DH-D3BJ	2.31	3.81	2.09	4.53	5.12	2.95	2.41	0.94	2.88	3.66
PBE0-DH	1.86	2.55	1.84	2.78	2.33	1.69	1.85	3.18	1.58	2.36
SOS0-PBE0-DH-D3BJ	1.98	2.98	1.55	3.72	3.64	2.69	1.74	1.26	2.29	2.90
SOS0-PBE0-DH	1.77	3.09	2.51	2.60	3.45	2.26	1.44	4.03	1.34	2.73
PBE-QIDH-D3BJ	2.72	3.67	1.63	3.19	4.90	2.14	1.84	1.30	2.19	3.21
PBE-QIDH	2.69	3.03	2.18	2.45	2.82	1.87	1.58	2.35	1.76	2.59
SOS1-PBE-QIDH-D3BJ	2.82	2.98	2.77	2.01	1.55	1.54	0.75	2.18	1.58	2.43
SOS1-PBE-QIDH	2.99	3.42	4.29	2.59	3.17	2.80	0.97	4.20	1.42	3.16
RSX-QIDH-D3BJ	4.32	6.32	4.21	1.90	1.90	3.23	2.33	4.02	3.60	4.41
RSX-QIDH	4.32	6.29	4.37	1.82	2.16	3.21	2.26	4.25	3.57	4.42
RSX-0DH-D3(BJ)	4.72	9.68	5.52	1.62	6.95	3.85	2.57	7.05	4.87	6.63
RSX-0DH	4.73	9.70	5.70	1.71	7.43	3.89	2.51	7.28	4.87	6.71
BLYP-D3BJ	7.07	14.18	14.35	11.49	15.18	6.96	4.24	10.08	7.31	12.10
BLYP	6.58	13.99	10.23	6.64	6.44	7.37	4.04	4.63	6.84	9.82
B3LYP-D3BJ	3.87	7.80	7.71	7.08	8.23	4.08	2.19	4.89	4.12	6.77
B3LYP	3.70	8.80	4.80	4.32	5.03	6.00	2.36	2.61	4.23	6.17
BH&HLYP-D3BJ	1.51	3.87	2.31	2.45	5.11	3.26	2.54	3.01	1.64	3.24
BH&HLYP	2.43	7.78	5.77	6.00	12.89	6.90	3.46	6.52	3.64	7.19
CAM-B3LYP-D3BJ	1.37	2.53	2.72	3.78	1.58	2.24	1.84	1.98	1.24	2.59
CAM-B3LYP	1.70	5.30	2.67	3.05	7.25	4.85	1.54	4.19	2.27	4.39
B2PLYP-D3BJ	2.26	6.78	5.21	5.45	8.73	2.39	1.64	3.85	3.27	5.67
B2PLYP	2.05	6.25	3.40	3.33	4.29	3.00	1.46	2.02	2.94	4.37
B2GP-PLYP-D3BJ	1.68	5.19	2.66	3.92	6.75	1.36	1.15	2.34	2.26	4.17
B2GP-PLYP	1.71	4.58	1.60	2.59	3.61	2.29	0.96	1.50	1.94	3.22
ωB2PLYP-D3BJ	2.05	2.85	1.28	2.43	1.54	1.94	2.49	2.32	1.82	2.31
ωB2PLYP	2.04	2.85	1.33	2.33	1.74	1.97	2.45	2.41	1.80	2.31
ωB2G-PLYP-D3BJ	2.49	2.30	1.83	2.31	1.24	1.88	2.08	1.86	1.67	2.12
ωB2GP-PLYP	2.49	2.30	1.83	2.30	1.24	1.88	2.08	1.86	1.66	2.12
DSD-PBEP86-D3BJ	2.60	4.22	2.05	3.97	7.81	1.75	1.53	2.29	2.26	4.04
revDSD-PBEP86-D3BJ	2.80	2.72	1.69	3.05	5.78	1.10	0.89	1.64	1.63	2.96

revDOD-PBEP86-D3BJ	2.87	2.01	1.70	2.82	5.07	0.99	0.72	1.43	1.41	2.58
noDispSD-PBEP86	2.73	5.52	2.31	4.09	8.57	2.06	2.16	2.48	2.58	4.70
xDSD$_{75}$-PBEP86-D3BJ	2.30	2.73	1.60	3.06	6.24	1.08	0.76	1.63	1.45	3.00
xDOD$_{75}$-PBEP86-D3BJ	2.46	1.69	1.58	2.77	5.31	0.93	0.59	1.36	1.16	2.48
xnoDispSD-PBEP86	2.25	4.92	2.25	3.91	8.66	1.81	1.67	2.39	2.21	4.41
ωDSD$_{20}$-PBEP86-D3BJ (ω=0.30)	1.11	1.12	2.09	3.26	1.47	1.25	1.68	1.72	1.07	1.90
ωDSD$_{40}$-PBEP86-D3BJ (ω=0.30)	1.34	1.17	1.49	2.98	2.34	0.99	1.36	1.39	0.94	1.83
ωDSD$_{50}$-PBEP86-D3BJ (ω=0.30)	1.69	1.25	1.17	2.74	2.71	0.96	1.15	1.30	0.86	1.82
ωDSD$_{60}$-PBEP86-D3BJ (ω=0.22)	1.65	1.61	1.25	2.93	3.88	0.92	1.00	1.15	1.08	2.14
ωDSD$_{60}$-PBEP86-D3BJ (ω=0.30)	2.27	1.39	1.38	2.53	3.03	1.13	0.92	1.28	0.80	1.92
ωDSD$_{69}$-PBEP86-D3BJ (ω=0.10)	1.77	2.37	1.52	3.13	5.47	0.96	0.87	1.53	1.45	2.71
ωDSD$_{69}$-PBEP86-D3BJ (ω=0.16)	2.00	2.16	1.37	3.00	5.03	1.02	0.87	1.30	1.25	2.54
ωDSD$_{69}$-PBEP86-D3BJ (ω=0.20)	2.24	1.87	1.39	2.82	4.50	1.07	0.83	1.14	1.04	2.35
ωDSD$_{69}$-PBEP86-D3BJ (ω=0.25)	2.62	1.61	1.73	2.62	3.86	1.23	0.78	1.13	0.85	2.19
ωDSD$_{69}$-PBEP86-D3BJ (ω=0.30)	3.05	1.68	2.18	2.57	3.68	1.46	0.79	1.31	0.82	2.28
ωDSD$_{72}$-PBEP86-D3BJ (ω=0.08)	2.02	2.48	1.50	3.08	5.74	1.00	0.81	1.54	1.43	2.80
ωDSD$_{72}$-PBEP86-D3BJ (ω=0.13)	2.16	2.30	1.44	2.99	5.34	1.05	0.81	1.34	1.28	2.66
ωDOD$_{20}$-PBEP86-D3BJ (ω=0.30)	1.07	1.23	1.98	3.09	1.28	1.23	1.56	1.73	0.97	1.83
ωDOD$_{40}$-PBEP86-D3BJ (ω=0.30)	1.44	1.46	1.23	2.58	1.42	0.94	1.08	1.44	0.77	1.66
ωDOD$_{50}$-PBEP86-D3BJ (ω=0.30)	1.88	1.63	1.15	2.28	1.51	0.94	0.82	1.39	0.72	1.66
ωDOD$_{60}$-PBEP86-D3BJ (ω=0.22)	1.77	1.00	1.06	2.55	2.82	0.79	0.74	0.99	0.81	1.71
ωDOD$_{60}$-PBEP86-D3BJ (ω=0.30)	2.54	1.74	1.67	2.21	1.98	1.15	0.68	1.36	0.74	1.86
ωDOD$_{69}$-PBEP86-D3BJ (ω=0.10)	1.85	1.60	1.29	2.85	4.65	0.79	0.68	1.28	1.21	2.27
ωDOD$_{69}$-PBEP86-D3BJ (ω=0.16)	2.15	1.12	1.28	2.57	3.73	0.84	0.61	0.97	0.89	1.95
ωDOD$_{69}$-PBEP86-D3BJ (ω=0.20)	2.44	1.09	1.54	2.45	3.30	0.97	0.58	0.98	0.75	1.90
ωDOD$_{69}$-PBEP86-D3BJ (ω=0.25)	2.90	1.50	2.10	2.29	2.60	1.23	0.59	1.16	0.74	1.98
ωDOD$_{69}$-PBEP86-D3BJ (ω=0.30)	3.41	1.98	2.70	2.23	2.19	1.51	0.60	1.45	0.85	2.22
ωDOD$_{72}$-PBEP86-D3BJ (ω=0.08)	2.13	1.54	1.34	2.77	4.75	0.83	0.62	1.24	1.14	2.29
ωDOD$_{72}$-PBEP86-D3BJ (ω=0.13)	2.33	1.24	1.43	2.62	4.18	0.89	0.58	1.04	0.95	2.11
ωnoDispSD$_{20}$-PBEP86 (ω=0.30)	1.99	5.23	5.27	6.18	6.88	2.65	4.56	2.75	2.86	5.04
ωnoDispSD$_{40}$-PBEP86 (ω=0.30)	1.61	4.03	3.41	4.74	6.16	2.06	3.29	2.14	2.11	3.94
ωnoDispSD$_{50}$-PBEP86 (ω=0.30)	1.76	3.48	2.39	4.03	5.76	1.86	2.65	1.88	1.74	3.43
ωnoDispSD$_{60}$-PBEP86 (ω=0.22)	1.71	4.49	2.75	4.32	7.32	1.97	2.49	2.13	2.15	4.12
ωnoDispSD$_{60}$-PBEP86 (ω=0.30)	2.24	3.31	1.82	3.57	5.93	1.85	2.10	1.79	1.51	3.28
ωnoDispSD$_{69}$-PBEP86 (ω=0.10)	1.79	5.07	2.81	4.31	8.45	1.91	2.10	2.45	2.40	4.52
ωnoDispSD$_{69}$-PBEP86 (ω=0.16)	1.97	4.52	2.26	3.98	7.63	1.87	2.00	2.07	2.07	4.08
ωnoDispSD$_{69}$-PBEP86 (ω=0.20)	2.20	4.05	1.97	3.75	7.10	1.86	1.90	1.88	1.82	3.77
ωnoDispSD$_{69}$-PBEP86 (ω=0.25)	2.58	3.29	1.75	3.34	6.06	1.83	1.68	1.64	1.42	3.25
ωnoDispSD$_{69}$-PBEP86 (ω=0.30)	3.02	2.99	1.98	3.16	5.66	1.93	1.54	1.65	1.22	3.11
ωnoDispSD$_{72}$-PBEP86 (ω=0.08)	1.99	5.00	2.52	4.14	8.54	1.87	1.91	2.41	2.31	4.46
ωnoDispSD$_{72}$-PBEP86 (ω=0.13)	2.12	4.55	2.17	3.89	7.85	1.84	1.82	2.11	2.06	4.11
PBE10	3.44	6.82	7.98	7.39	7.81	3.50	4.88	2.96	4.27	6.43
PBE0	1.87	3.47	3.63	4.43	3.65	2.27	3.05	2.51	2.24	3.42
PBE32	1.67	3.01	2.52	3.46	3.52	2.25	2.33	3.70	1.64	2.90
PBE38	1.84	3.58	2.81	3.06	4.61	2.50	1.85	4.75	1.60	3.23
PBE50	2.78	6.37	5.55	3.67	8.57	3.57	1.42	7.16	2.80	5.40
PBE60	3.67	9.04	8.06	5.02	12.11	4.61	1.92	8.99	4.17	7.57
r2SCAN	3.63	8.38	9.35	8.57	11.42	3.66	4.59	5.24	4.99	7.90
r2SCANh	2.63	6.01	6.48	6.44	8.06	2.56	3.60	3.04	3.74	5.69
r2SCAN0	1.60	3.10	2.76	3.69	3.39	1.51	2.31	1.62	2.09	2.91
r2SCAN30	1.49	2.68	2.04	3.00	2.57	1.50	1.96	2.26	1.71	2.43
r2SCAN32	1.48	2.66	1.95	2.79	2.54	1.56	1.84	2.58	1.62	2.37
r2SCAN35	1.52	2.80	2.07	2.55	2.86	1.69	1.68	3.09	1.55	2.43
r2SCAN50	2.20	5.32	4.79	3.12	7.15	2.82	1.46	5.78	2.44	4.52
r2SCAN60	2.82	7.54	6.87	4.47	10.51	3.72	1.84	7.51	3.52	6.42
TPSS	5.00	10.71	9.41	6.92	6.96	5.49	4.20	5.30	5.42	8.15
TPSSh	3.61	7.93	6.36	5.25	4.68	4.51	3.14	3.24	3.98	5.96
TPSS0	1.95	4.51	2.98	3.75	4.52	3.62	1.93	2.84	2.04	3.73

TPSS30	1.63	3.92	2.81	3.66	5.54	3.54	1.71	3.54	1.64	3.61
TPSS38	1.58	4.07	3.81	3.94	7.52	3.65	1.64	4.87	1.63	4.16
TPSS45	1.98	5.29	5.44	4.59	9.73	3.99	1.88	6.28	2.30	5.29
TPSS50	2.37	6.42	6.61	5.14	11.26	4.31	2.14	7.22	2.92	6.21
TPSS60	3.25	8.99	8.93	6.35	14.37	5.10	2.79	9.06	4.27	8.21
PBE0-D3BJ	2.49	4.39	5.49	6.95	8.25	3.71	3.64	2.14	3.58	5.28
PBE38-D3BJ	2.11	3.27	2.04	4.29	3.81	2.71	2.35	1.64	2.60	3.23
r2SCAN-D3BJ	3.96	9.07	10.65	10.19	14.91	4.99	4.97	6.84	5.58	9.25
r2SCANh-D3BJ	2.99	6.74	7.81	8.09	11.64	3.98	4.00	4.60	4.39	7.08
r2SCAN0-D3BJ	1.97	3.76	3.88	5.20	6.64	2.82	2.74	1.76	2.84	4.14
r2SCAN50-D3BJ	2.24	4.84	3.22	2.16	3.41	2.75	1.69	3.86	2.60	3.52
TPSS-D3BJ	5.71	11.91	12.47	10.58	14.18	6.97	4.73	9.22	6.52	10.65
TPSSh-D3BJ	4.29	8.92	9.20	8.44	10.96	5.56	3.55	6.57	5.01	8.12
TPSS0-D3BJ	2.63	4.88	4.58	5.45	5.72	3.74	2.06	3.09	3.04	4.61
B97-1	2.87	5.66	5.11	4.82	3.76	2.97	2.47	1.77	2.98	4.56
B97-D3BJ	6.47	13.19	11.98	10.84	15.21	6.96	4.28	8.29	6.91	11.27
B97	6.02	13.23	7.85	6.02	6.36	7.70	4.35	3.79	6.53	9.13
B97-D4	6.11	12.42	10.68	8.55	10.86	5.90	4.11	6.26	6.43	9.77
PBE-D4	5.64	11.04	13.96	13.05	17.15	7.18	6.97	8.61	7.15	11.50
revPBE	4.92	10.65	8.53	6.75	6.93	6.13	5.18	4.02	5.58	8.03
revPBE-D3BJ	5.81	11.98	12.53	11.80	16.48	7.20	5.43	8.01	6.99	11.24
revPBE-D4	6.10	12.44	13.02	12.08	16.16	7.71	5.92	8.75	7.60	11.55
PBE0-D4	2.56	4.50	5.57	7.01	8.10	3.73	3.74	2.23	3.69	5.32
B3LYP-D4	3.84	7.69	7.77	7.08	7.95	4.01	2.33	4.94	4.15	6.70
ωB97X-D3(0)	1.29	3.37	1.72	2.98	1.87	2.11	0.87	3.18	1.39	2.63
ωB97X-D4	1.49	3.47	1.27	2.56	4.35	2.54	0.94	2.86	1.82	2.87
PWPB95	1.52	3.25	2.15	2.70	2.28	2.07	1.49	1.85	1.75	2.57
PWPB95-D3BJ	1.80	3.49	3.21	3.92	4.81	2.62	1.15	1.53	1.97	3.35
PWPB95-D4	1.87	3.54	3.33	4.12	4.98	2.65	1.24	1.77	2.18	3.47
$xDSD_{75}$-PBEP86-D4	2.30	2.16	1.45	2.65	4.97	1.00	0.61	1.31	1.22	2.48
$xDOD_{75}$-PBEP86-D4	2.45	1.38	1.55	2.43	4.18	1.00	0.55	1.11	0.98	2.11
ωDOD_{69}-PBEP86-D4 (ω=0.10)	1.84	1.64	1.35	2.87	4.62	0.80	0.75	1.42	1.31	2.29
ωDOD_{60}-PBEP86-D4 (ω=0.22)	1.73	1.06	1.15	2.58	2.82	0.79	0.83	1.05	1.01	1.74
HF-PBE	5.83	9.37	10.89	10.28	8.42	6.79	3.83	6.11	4.37	8.74
HF-PBE-D4	4.51	11.06	8.81	7.45	15.38	4.48	4.53	3.26	5.33	9.22
HF-PBE0	5.55	3.91	11.47	9.32	3.73	5.68	2.07	7.23	1.73	6.63
HF-PBE0-D4	4.65	4.98	9.01	5.84	6.47	2.83	2.53	3.12	2.59	5.48

Table S3: Root-mean-square deviations (RMSDs, kcal/mol) for different DFT functionals on the complete BH9 reaction energy set and its nine subsets. The subsets (or the reaction types) of the BH9 are radical rearrangement (I), Diels-Alder (II), halogen atom transfer (III), hydrogen atom transfer (IV), hydride transfer (V), Boron and Silicon containing reactions (VI), proton transfer (VII), nucleophilic substitution (VIII), and nucleophilic addition (IX). Heatmapping is from red (worst) via yellow to green (best).

	RMSD (kcal/mol)									
**Functionals**	**I**	**II**	**III**	**IV**	**V**	**VI**	**VII**	**VIII**	**IX**	**Total**
B97M-V	2.13	6.14	4.55	2.72	3.39	2.11	2.59	2.37	3.06	4.25
BMK-D3BJ	3.00	3.71	2.09	1.99	2.79	5.34	1.60	1.44	2.69	3.16
BMK	2.09	4.53	2.48	2.14	3.16	2.53	1.67	1.57	2.08	3.19
ωB97X-D	1.49	2.31	2.61	1.93	1.23	3.03	1.40	1.61	1.64	2.11
ωB97X-V	2.66	4.23	1.50	2.20	1.10	3.50	1.22	1.58	2.49	3.01
ωB97M-V	1.36	1.76	1.76	1.52	1.59	2.44	1.10	1.50	1.47	1.69
ωB97X-2-D3BJ	3.51	1.34	2.49	2.76	0.86	1.64	0.74	1.05	1.11	2.10
ωB97X-2	3.51	1.35	2.49	2.76	0.86	1.64	0.74	1.05	1.11	2.10
ωB97M(2)	1.39	1.73	0.98	1.44	0.66	1.70	0.62	0.91	1.40	1.44
PBE-D3BJ	2.32	6.35	6.35	3.44	3.65	1.73	3.33	3.19	4.28	4.75
PBE	3.24	10.60	6.71	3.60	3.55	5.24	3.31	3.08	3.79	6.91
PBE20	2.47	6.20	4.34	2.47	2.00	3.53	2.17	2.02	2.47	4.20
LRC-ωPBEh	3.25	3.96	3.31	2.16	1.55	2.93	1.48	1.63	1.87	3.04
PBE0-2-D3BJ	6.21	5.44	3.47	3.66	0.91	3.27	0.79	0.86	3.81	4.36
PBE0-2	6.06	4.78	3.44	3.64	0.93	2.39	0.78	0.74	3.25	4.00
SOS0-PBE0-2-D3BJ	5.17	3.99	3.40	3.25	0.86	2.21	0.58	0.68	2.32	3.44
SOS0-PBE0-2	5.00	2.33	3.37	3.25	0.99	1.78	0.56	0.86	0.99	2.83
PBE0-DH-D3BJ	4.08	5.70	2.02	1.70	1.48	3.87	1.24	1.51	4.77	3.96
PBE0-DH	3.23	3.84	2.43	1.73	1.64	1.46	1.19	1.23	2.49	2.78
SOS0-PBE0-DH-D3BJ	3.44	4.75	1.99	1.61	1.53	3.76	1.12	1.49	3.92	3.39
SOS0-PBE0-DH	2.79	3.53	2.44	1.67	1.74	1.84	1.09	1.26	1.82	2.58
PBE-QIDH-D3BJ	4.36	5.35	1.57	2.20	1.13	2.93	0.85	0.94	3.84	3.72
PBE-QIDH	4.13	4.62	1.61	2.19	1.19	1.72	0.83	0.87	3.12	3.26
SOS1-PBE-QIDH-D3BJ	3.45	4.35	1.48	1.94	1.19	2.49	0.67	0.92	2.79	3.04
SOS1-PBE-QIDH	3.05	3.00	1.67	1.97	1.34	1.44	0.65	0.98	1.40	2.31
RSX-QIDH-D3BJ	6.57	11.05	1.52	1.90	1.55	4.33	0.83	1.53	6.38	6.91
RSX-QIDH	6.53	10.93	1.52	1.90	1.55	4.10	0.83	1.50	6.27	6.83
RSX-0DH-D3(BJ)	7.67	13.38	1.80	1.35	2.28	4.74	1.01	2.20	7.24	8.26
RSX-0DH	7.65	13.26	1.81	1.35	2.28	4.51	1.01	2.18	7.13	8.18
BLYP-D3BJ	6.10	14.48	6.13	3.20	3.05	3.34	2.99	3.30	5.20	8.88
BLYP	8.92	23.33	6.82	3.65	3.03	12.02	3.01	3.26	10.25	14.29
B3LYP-D3BJ	3.72	9.04	3.58	2.15	2.64	2.00	1.88	2.31	3.38	5.57
B3LYP	6.04	16.45	4.32	2.50	2.96	8.74	1.90	2.22	7.44	10.08
BH&HLYP-D3BJ	1.88	4.10	1.99	2.06	4.06	2.77	1.03	2.37	1.79	3.07
BH&HLYP	3.46	9.64	2.67	2.24	4.36	5.47	1.06	2.24	4.59	6.12
CAM-B3LYP-D3BJ	1.90	4.16	2.31	1.53	2.30	1.90	1.13	2.13	1.93	2.82
CAM-B3LYP	2.98	8.19	2.73	1.69	2.58	5.00	1.13	1.76	3.90	5.17
B2PLYP-D3BJ	2.33	5.70	1.78	1.80	1.81	1.14	1.19	1.47	2.20	3.53
B2PLYP	3.20	9.27	2.09	1.90	1.95	4.39	1.19	1.43	4.03	5.65
B2GP-PLYP-D3BJ	2.51	3.50	1.60	2.09	1.72	1.01	0.84	1.19	1.53	2.49
B2GP-PLYP	2.83	6.02	1.72	2.13	1.82	2.88	0.83	1.10	2.55	3.84
ωB2PLYP-D3BJ	2.58	3.85	1.27	1.32	2.00	2.29	0.74	1.71	3.02	2.69
ωB2PLYP	2.55	3.79	1.27	1.32	2.00	2.16	0.74	1.69	2.95	2.65
ωB2G-PLYP-D3BJ	3.03	3.74	1.56	1.76	1.93	2.24	0.69	1.43	2.95	2.74
ωB2GP-PLYP	3.03	3.74	1.56	1.76	1.93	2.23	0.69	1.43	2.95	2.74
DSD-PBEP86-D3BJ	3.83	1.42	2.39	2.82	0.89	1.73	0.80	1.09	1.86	2.21
revDSD-PBEP86-D3BJ	3.74	1.46	2.45	2.76	0.83	1.20	0.76	0.95	1.15	2.14

revDOD-PBEP86-D3BJ	3.62	1.30	2.40	2.69	0.84	1.10	0.77	0.92	1.11	2.05
noDispSD-PBEP86	4.32	2.29	2.73	3.30	1.03	1.91	0.99	1.22	1.46	2.67
xDSD$_{75}$-PBEP86-D3BJ	3.09	1.39	2.14	2.50	0.76	1.16	0.62	0.75	1.01	1.89
xDOD$_{75}$-PBEP86-D3BJ	3.00	1.17	2.13	2.44	0.76	1.03	0.70	0.69	0.98	1.80
xnoDispSD-PBEP86	3.50	1.99	2.39	2.92	0.99	1.65	0.72	1.03	1.25	2.29
ωDSD$_{20}$-PBEP86-D3BJ (ω=0.30)	1.18	1.41	1.85	1.66	1.39	1.54	1.28	1.99	1.19	1.50
ωDSD$_{40}$-PBEP86-D3BJ (ω=0.30)	1.63	1.57	1.40	1.61	1.14	1.48	1.01	1.49	1.07	1.49
ωDSD$_{50}$-PBEP86-D3BJ (ω=0.30)	2.08	1.79	1.40	1.78	1.01	1.46	0.87	1.21	1.10	1.64
ωDSD$_{60}$-PBEP86-D3BJ (ω=0.22)	2.06	1.27	1.39	1.86	0.80	1.20	0.81	0.99	1.01	1.45
ωDSD$_{60}$-PBEP86-D3BJ (ω=0.30)	2.76	2.09	1.78	2.13	0.89	1.50	0.74	0.94	1.20	1.94
ωDSD$_{69}$-PBEP86-D3BJ (ω=0.10)	2.32	1.40	1.55	2.05	0.74	1.01	0.75	0.83	1.02	1.57
ωDSD$_{69}$-PBEP86-D3BJ (ω=0.16)	2.60	1.23	1.75	2.20	0.72	1.17	0.70	0.81	1.00	1.64
ωDSD$_{69}$-PBEP86-D3BJ (ω=0.20)	2.84	1.35	1.93	2.31	0.74	1.29	0.67	0.78	1.03	1.77
ωDSD$_{69}$-PBEP86-D3BJ (ω=0.25)	3.21	1.85	2.18	2.45	0.77	1.46	0.64	0.75	1.17	2.04
ωDSD$_{69}$-PBEP86-D3BJ (ω=0.30)	3.68	2.35	2.53	2.68	0.83	1.64	0.63	0.71	1.32	2.37
ωDSD$_{72}$-PBEP86-D3BJ (ω=0.08)	2.68	1.37	1.81	2.25	0.74	1.08	0.69	0.78	1.01	1.71
ωDSD$_{72}$-PBEP86-D3BJ (ω=0.13)	2.84	1.23	1.93	2.34	0.70	1.18	0.66	0.76	1.01	1.74
ωDOD$_{20}$-PBEP86-D3BJ (ω=0.30)	1.13	1.43	1.84	1.61	1.48	1.57	1.29	1.97	1.16	1.50
ωDOD$_{40}$-PBEP86-D3BJ (ω=0.30)	1.54	1.81	1.35	1.51	1.25	1.52	1.08	1.47	1.06	1.55
ωDOD$_{50}$-PBEP86-D3BJ (ω=0.30)	1.94	2.04	1.33	1.67	1.14	1.46	0.98	1.21	1.06	1.69
ωDOD$_{60}$-PBEP86-D3BJ (ω=0.22)	1.95	1.33	1.34	1.78	0.87	1.16	0.88	0.98	0.97	1.43
ωDOD$_{60}$-PBEP86-D3BJ (ω=0.30)	2.65	2.42	1.78	2.07	1.07	1.49	0.91	0.96	1.17	2.03
ωDOD$_{69}$-PBEP86-D3BJ (ω=0.10)	2.23	1.24	1.52	1.98	0.76	0.94	0.80	0.80	0.99	1.49
ωDOD$_{69}$-PBEP86-D3BJ (ω=0.16)	2.46	1.20	1.68	2.09	0.74	1.07	0.78	0.77	0.97	1.57
ωDOD$_{69}$-PBEP86-D3BJ (ω=0.20)	2.72	1.51	1.90	2.22	0.81	1.20	0.78	0.76	1.00	1.76
ωDOD$_{69}$-PBEP86-D3BJ (ω=0.25)	3.10	2.28	2.16	2.37	0.90	1.42	0.80	0.75	1.22	2.14
ωDOD$_{69}$-PBEP86-D3BJ (ω=0.30)	3.54	2.86	2.51	2.57	1.00	1.58	0.86	0.74	1.37	2.50
ωDOD$_{72}$-PBEP86-D3BJ (ω=0.08)	2.56	1.16	1.77	2.17	0.75	0.98	0.75	0.74	0.98	1.61
ωDOD$_{72}$-PBEP86-D3BJ (ω=0.13)	2.71	1.16	1.89	2.25	0.73	1.06	0.75	0.72	0.97	1.67
ωnoDispSD$_{20}$-PBEP86 (ω=0.30)	2.28	2.36	2.61	2.66	2.25	1.87	1.66	2.06	1.97	2.35
ωnoDispSD$_{40}$-PBEP86 (ω=0.30)	2.46	1.99	2.00	2.40	1.64	1.64	1.18	1.50	1.62	2.03
ωnoDispSD$_{50}$-PBEP86 (ω=0.30)	2.77	2.01	1.89	2.44	1.37	1.60	0.96	1.21	1.53	2.05
ωnoDispSD$_{60}$-PBEP86 (ω=0.22)	2.67	1.78	1.85	2.53	1.23	1.63	0.95	1.15	1.40	1.97
ωnoDispSD$_{60}$-PBEP86 (ω=0.30)	3.34	1.99	2.23	2.75	1.24	1.71	0.77	1.01	1.42	2.23
ωnoDispSD$_{69}$-PBEP86 (ω=0.10)	2.78	2.11	1.86	2.58	1.03	1.57	0.90	1.09	1.36	2.08
ωnoDispSD$_{69}$-PBEP86 (ω=0.16)	3.06	1.74	2.05	2.70	1.02	1.64	0.81	1.00	1.31	2.07
ωnoDispSD$_{69}$-PBEP86 (ω=0.20)	3.34	1.64	2.26	2.83	1.05	1.69	0.74	0.95	1.29	2.14
ωnoDispSD$_{69}$-PBEP86 (ω=0.25)	3.68	1.79	2.50	2.92	1.03	1.72	0.65	0.84	1.32	2.29
ωnoDispSD$_{69}$-PBEP86 (ω=0.30)	4.13	2.09	2.86	3.12	1.08	1.83	0.62	0.79	1.38	2.54
ωnoDispSD$_{72}$-PBEP86 (ω=0.08)	3.12	2.01	2.09	2.74	1.01	1.63	0.81	1.06	1.32	2.17
ωnoDispSD$_{72}$-PBEP86 (ω=0.13)	3.28	1.77	2.22	2.81	0.99	1.68	0.76	0.99	1.27	2.15
PBE10	2.65	8.25	5.34	2.98	2.48	4.37	2.70	2.53	3.06	5.43
PBE0	2.54	5.40	3.99	2.28	1.93	3.15	1.95	1.82	2.26	3.74
PBE32	2.83	4.58	3.52	2.03	1.99	2.63	1.61	1.61	2.14	3.28
PBE38	3.16	4.40	3.24	1.91	2.16	2.32	1.40	1.53	2.21	3.18
PBE50	4.13	5.55	2.82	1.88	2.74	2.11	1.09	1.70	2.85	3.81
PBE60	5.06	7.40	2.73	2.11	3.33	2.59	1.09	2.11	3.68	4.89
r2SCAN	1.80	6.89	4.94	2.48	2.30	2.11	2.61	2.34	3.16	4.52
r2SCANh	1.71	5.42	4.11	2.12	2.09	1.86	2.19	2.01	2.73	3.65
r2SCAN0	2.12	3.89	3.38	1.77	2.32	1.83	1.64	1.70	2.40	2.86
r2SCAN30	2.35	3.71	3.22	1.72	2.48	1.91	1.48	1.66	2.40	2.79
r2SCAN32	2.45	3.69	3.17	1.70	2.55	1.96	1.42	1.66	2.41	2.80
r2SCAN35	2.61	3.74	3.09	1.70	2.66	2.04	1.34	1.66	2.45	2.84
r2SCAN50	3.52	4.89	2.87	1.87	3.27	2.63	1.06	1.90	2.90	3.53
r2SCAN60	4.20	6.17	2.87	2.15	3.71	3.13	1.03	2.19	3.39	4.29
TPSS	4.24	14.24	6.38	3.66	3.33	7.82	2.53	3.19	5.34	8.92
TPSSh	3.37	11.56	5.12	3.20	2.32	6.75	2.09	2.78	4.42	7.28
TPSS0	2.53	7.81	3.85	2.73	1.69	5.15	1.54	2.29	2.98	5.07

TPSS30	2.44	6.70	3.52	2.64	1.69	4.63	1.40	2.18	2.51	4.44
TPSS38	2.58	5.34	3.17	2.56	1.89	3.92	1.25	2.08	1.91	3.71
TPSS45	2.98	4.62	2.92	2.54	2.25	3.33	1.18	2.10	1.58	3.37
TPSS50	3.35	4.65	2.83	2.57	2.54	3.05	1.18	2.17	1.64	3.42
TPSS60	4.27	5.87	2.79	2.73	3.19	2.89	1.28	2.44	2.34	4.13
PBE0-D3BJ	2.78	3.31	3.56	2.17	1.76	2.59	1.97	1.95	3.93	2.89
PBE38-D3BJ	3.73	5.03	2.81	1.84	1.91	3.63	1.42	1.69	4.38	3.66
r2SCAN-D3BJ	1.74	5.37	4.83	2.44	2.31	2.44	2.64	2.49	3.72	3.86
r2SCANh-D3BJ	1.80	3.96	3.98	2.08	2.04	2.71	2.22	2.18	3.45	3.09
r2SCAN0-D3BJ	2.38	3.17	3.23	1.75	2.22	3.36	1.66	1.89	3.44	2.75
r2SCAN50-D3BJ	3.91	5.96	2.68	1.87	3.15	4.79	1.07	2.08	4.27	4.25
TPSS-D3BJ	2.53	8.42	5.95	3.45	3.54	2.86	2.57	2.92	3.36	5.61
TPSSh-D3BJ	1.95	6.01	4.66	3.01	2.43	2.50	2.13	2.50	2.79	4.17
TPSS0-D3BJ	2.15	2.94	3.28	2.59	1.61	2.86	1.58	2.01	2.74	2.66
B97-1	3.10	9.72	4.21	2.52	2.45	4.34	1.97	1.76	3.50	6.03
B97-D3BJ	4.92	12.34	5.99	2.97	3.08	2.80	3.28	2.77	4.49	7.64
B97	8.57	22.84	6.92	3.50	2.96	12.63	3.33	3.42	10.34	14.06
B97-D4	5.50	14.65	6.24	3.12	2.78	5.40	3.25	2.91	5.48	9.01
PBE-D4	2.31	5.91	6.33	3.45	3.46	1.72	3.29	3.29	4.45	4.56
revPBE	5.50	16.64	7.20	3.78	3.87	10.35	3.36	3.91	7.17	10.53
revPBE-D3BJ	2.24	6.96	6.38	3.36	4.10	2.18	3.34	3.42	3.68	5.01
revPBE-D4	1.93	4.87	6.20	3.30	3.42	2.00	3.13	3.55	4.43	4.10
PBE0-D4	2.91	3.50	3.61	2.17	1.82	2.52	1.95	1.94	4.10	2.98
B3LYP-D4	3.48	8.54	3.64	2.14	2.93	2.03	1.83	2.20	3.19	5.31
ωB97X-D3(0)	1.79	2.07	2.32	2.00	1.25	2.33	1.35	1.59	1.53	1.96
ωB97X-D4	2.43	4.28	1.77	2.19	1.11	3.92	1.24	2.03	2.92	3.10
PWPB95	1.68	5.10	2.29	1.82	1.50	2.84	1.20	1.39	2.32	3.29
PWPB95-D3BJ	1.34	3.10	2.13	1.76	1.40	1.22	1.21	1.28	1.51	2.18
PWPB95-D4	1.39	2.35	2.08	1.73	1.59	1.11	1.16	1.12	1.45	1.86
xDSD$_{75}$-PBEP86-D4	2.94	1.49	2.04	2.35	0.76	1.07	0.59	0.64	0.92	1.83
xDOD$_{75}$-PBEP86-D4	2.88	1.35	2.05	2.32	0.76	0.95	0.68	0.60	0.87	1.77
ωDOD$_{69}$-PBEP86-D4 (ω=0.10)	2.24	1.21	1.54	1.99	0.73	0.83	0.77	0.71	1.01	1.49
ωDOD$_{60}$-PBEP86-D4 (ω=0.22)	1.98	1.41	1.38	1.78	0.72	0.98	0.85	0.87	1.08	1.44
HF-PBE	5.88	10.60	8.39	9.44	1.83	9.91	2.57	3.85	4.78	8.56
HF-PBE-D4	4.91	4.60	8.39	9.39	1.71	5.72	2.48	3.44	2.53	6.11
HF-PBE0	5.24	5.63	5.94	6.58	1.65	6.12	1.57	2.28	2.13	5.34
HF-PBE0-D4	5.24	4.06	5.91	6.58	1.57	3.71	1.53	1.91	3.08	4.71

Table S4: Mean signed deviations (MSDs, kcal/mol) for different DFT functionals on the complete BH9 barrier height set and its nine subsets. The subsets (or the reaction types) of the BH9 are radical rearrangement (I), Diels-Alder (II), halogen atom transfer (III), hydrogen atom transfer (IV), hydride transfer (V), Boron and Silicon containing reactions (VI), proton transfer (VII), nucleophilic substitution (VIII), and nucleophilic addition (IX). A negative (red) value means underestimation, and a positive (blue) value represents the overestimation of barrier height.

	MSD (kcal/mol)									
**Functionals**	**I**	**II**	**III**	**IV**	**V**	**VI**	**VII**	**VIII**	**IX**	**Total**
B97M-V	-2.32	-4.54	-7.35	-6.18	-9.99	-2.58	-0.77	-3.59	-2.67	-5.04
BMK-D3BJ	-0.92	-0.07	-2.38	-4.25	-3.01	-2.51	0.33	-0.21	-0.51	-1.71
BMK	-0.07	2.83	1.40	0.11	4.62	2.00	1.32	4.09	0.99	1.84
ωB97X-D	-0.18	1.17	-0.55	-2.96	-1.57	-0.67	-0.29	1.52	0.05	-0.45
ωB97X-V	0.64	3.47	0.56	-1.89	2.97	0.17	-0.34	2.26	0.97	1.24
ωB97M-V	0.00	0.97	-0.70	-1.68	2.53	-0.89	-0.29	0.48	0.04	0.07
ωB97X-2-D3BJ	1.04	-3.96	-1.51	-2.49	-4.46	-0.71	-1.33	-1.47	-1.67	-2.42
ωB97X-2	1.04	-3.96	-1.50	-2.49	-4.45	-0.70	-1.33	-1.47	-1.67	-2.41
ωB97M(2)	-0.28	-0.65	-1.72	-2.30	-1.07	-0.68	-0.47	-0.45	-0.38	-1.06
PBE-D3BJ	-5.22	-10.01	-12.74	-12.02	-16.10	-6.35	-6.11	-7.80	-6.28	-10.07
PBE	-4.52	-7.64	-9.79	-8.41	-9.61	-2.92	-5.21	-4.20	-5.04	-7.16
PBE20	-1.71	-2.41	-3.57	-3.82	-3.22	-0.46	-2.84	0.63	-2.24	-2.55
LRC-ωPBEh	0.33	2.08	1.63	-0.58	3.49	1.38	-1.74	4.90	0.21	1.30
PBE0-2-D3BJ	3.01	-3.19	1.28	-2.17	-6.83	-0.81	-1.67	0.48	-1.22	-1.78
PBE0-2	3.15	-2.71	2.09	-1.32	-5.13	0.10	-1.46	1.36	-0.96	-1.09
SOS0-PBE0-2-D3BJ	4.10	1.31	4.06	0.21	-1.94	1.04	0.28	1.83	0.32	1.26
SOS0-PBE0-2	4.47	2.58	5.63	2.20	1.69	2.84	0.81	3.83	0.99	2.85
PBE0-DH-D3BJ	-0.32	-1.38	-1.67	-3.83	-4.84	-2.08	-1.90	0.13	-1.32	-2.12
PBE0-DH	0.24	0.50	0.68	-1.14	-0.12	0.62	-1.25	2.88	-0.34	0.11
SOS0-PBE0-DH-D3BJ	0.10	0.11	-0.88	-2.93	-3.37	-1.71	-1.24	0.73	-0.77	-1.12
SOS0-PBE0-DH	0.68	2.07	1.79	0.04	1.87	1.46	-0.57	3.70	0.24	1.31
PBE-QIDH-D3BJ	1.26	-1.45	0.50	-2.28	-4.63	-0.83	-1.49	1.09	-0.81	-1.27
PBE-QIDH	1.44	-0.84	1.50	-1.20	-2.51	0.29	-1.22	2.20	-0.48	-0.40
SOS1-PBE-QIDH-D3BJ	2.06	1.63	2.23	-0.55	-1.23	0.28	-0.08	2.06	0.25	0.82
SOS1-PBE-QIDH	2.43	2.89	3.95	1.39	2.29	2.25	0.38	4.01	0.91	2.40
RSX-QIDH-D3BJ	2.61	2.56	3.61	-0.85	0.45	0.43	-1.65	3.71	1.09	1.48
RSX-QIDH	2.63	2.63	3.80	-0.68	0.90	0.60	-1.59	3.93	1.14	1.62
RSX-0DH-D3(BJ)	2.54	6.69	5.16	0.25	5.59	1.45	-1.61	6.41	2.64	3.87
RSX-0DH	2.56	6.77	5.35	0.43	6.05	1.61	-1.55	6.63	2.69	4.02
BLYP-D3BJ	-5.97	-11.66	-13.18	-10.37	-13.97	-5.15	-3.48	-9.51	-6.37	-10.09
BLYP	-4.59	-7.03	-7.78	-3.76	-2.75	1.28	-1.91	-2.98	-3.98	-4.71
B3LYP-D3BJ	-3.00	-5.82	-7.08	-6.04	-7.54	-2.54	-1.49	-4.53	-3.44	-5.31
B3LYP	-1.86	-1.99	-2.53	-0.51	1.94	2.85	-0.17	0.96	-1.46	-0.82
BH&HLYP-D3BJ	0.54	2.62	1.78	0.77	4.07	1.51	2.01	1.34	1.00	1.84
BH&HLYP	1.42	5.57	5.38	5.04	11.48	5.73	3.03	5.62	2.53	5.34
CAM-B3LYP-D3BJ	-0.28	0.58	-2.02	-2.84	-0.51	0.11	-1.03	-0.03	-0.40	-0.70
CAM-B3LYP	0.37	2.76	0.97	0.68	5.96	3.58	-0.19	3.44	0.75	2.12
B2PLYP-D3BJ	-1.43	-5.70	-4.96	-4.48	-7.99	-1.59	-1.26	-3.32	-2.88	-4.48
B2PLYP	-0.87	-3.82	-2.63	-1.71	-3.15	1.15	-0.60	-0.57	-1.90	-2.23
B2GP-PLYP-D3BJ	0.00	-4.38	-2.40	-2.87	-6.15	-0.66	-0.76	-1.80	-1.97	-2.99
B2GP-PLYP	0.40	-3.05	-0.71	-0.96	-2.82	1.30	-0.32	0.14	-1.29	-1.41
ωB2PLYP-D3BJ	1.39	1.86	0.55	-1.72	0.64	1.03	-1.46	1.37	0.55	0.62
ωB2PLYP	1.40	1.91	0.67	-1.61	0.93	1.12	-1.42	1.51	0.58	0.71
ωB2G-PLYP-D3BJ	1.80	0.77	1.09	-1.39	-0.36	0.95	-1.12	1.12	0.30	0.33
ωB2GP-PLYP	1.80	0.77	1.10	-1.38	-0.34	0.96	-1.12	1.13	0.30	0.34
DSD-PBEP86-D3BJ	1.44	-3.69	-1.20	-2.89	-7.25	-0.97	-1.25	-1.66	-1.95	-2.64
revDSD-PBEP86-D3BJ	1.89	-2.24	-0.19	-1.78	-5.30	-0.08	-0.59	-0.91	-1.41	-1.50

revDOD-PBEP86-D3BJ	2.05	-1.49	0.15	-1.49	-4.62	0.11	-0.35	-0.70	-1.17	-1.05
noDispSD-PBEP86	1.37	-4.97	-1.49	-2.96	-7.91	-0.52	-1.89	-1.29	-2.35	-3.14
xDSD$_{75}$-PBEP86-D3BJ	1.39	-2.26	-0.45	-1.88	-5.69	-0.13	-0.50	-0.83	-1.26	-1.63
xDOD$_{75}$-PBEP86-D3BJ	1.70	-1.13	0.01	-1.53	-4.79	0.12	-0.19	-0.57	-0.94	-0.99
xnoDispSD-PBEP86	0.95	-4.41	-1.56	-2.85	-7.94	-0.55	-1.46	-1.25	-2.01	-2.97
ωDSD$_{20}$-PBEP86-D3BJ (ω=0.30)	0.00	-0.32	-1.25	-2.51	-1.05	-0.64	-1.35	0.40	-0.70	-0.93
ωDSD$_{40}$-PBEP86-D3BJ (ω=0.30)	0.49	-0.49	-0.85	-2.23	-2.12	-0.38	-1.08	0.36	-0.64	-0.91
ωDSD$_{50}$-PBEP86-D3BJ (ω=0.30)	0.93	-0.49	-0.33	-1.92	-2.47	-0.13	-0.90	0.50	-0.53	-0.74
ωDSD$_{60}$-PBEP86-D3BJ (ω=0.22)	0.85	-1.19	-0.67	-2.08	-3.58	-0.29	-0.76	-0.18	-0.88	-1.19
ωDSD$_{60}$-PBEP86-D3BJ (ω=0.30)	1.54	-0.46	0.42	-1.50	-2.75	0.18	-0.68	0.70	-0.38	-0.50
ωDSD$_{69}$-PBEP86-D3BJ (ω=0.10)	0.88	-1.93	-1.03	-2.17	-5.01	-0.34	-0.60	-0.88	-1.26	-1.65
ωDSD$_{69}$-PBEP86-D3BJ (ω=0.16)	1.15	-1.76	-0.54	-2.01	-4.62	-0.21	-0.65	-0.47	-1.07	-1.42
ωDSD$_{69}$-PBEP86-D3BJ (ω=0.20)	1.43	-1.42	-0.02	-1.76	-4.13	-0.03	-0.61	-0.07	-0.83	-1.10
ωDSD$_{69}$-PBEP86-D3BJ (ω=0.25)	1.84	-0.92	0.65	-1.42	-3.51	0.22	-0.54	0.43	-0.52	-0.65
ωDSD$_{69}$-PBEP86-D3BJ (ω=0.30)	2.24	-0.72	1.14	-1.20	-3.32	0.42	-0.56	0.78	-0.34	-0.40
ωDSD$_{72}$-PBEP86-D3BJ (ω=0.08)	1.14	-2.02	-0.74	-2.03	-5.25	-0.22	-0.55	-0.82	-1.24	-1.61
ωDSD$_{72}$-PBEP86-D3BJ (ω=0.13)	1.30	-1.88	-0.39	-1.91	-4.90	-0.14	-0.58	-0.52	-1.09	-1.43
ωDOD$_{20}$-PBEP86-D3BJ (ω=0.30)	0.17	0.36	-1.07	-2.36	-0.64	-0.52	-1.21	0.53	-0.54	-0.59
ωDOD$_{40}$-PBEP86-D3BJ (ω=0.30)	0.84	0.79	-0.32	-1.81	-1.07	-0.09	-0.74	0.71	-0.26	-0.17
ωDOD$_{50}$-PBEP86-D3BJ (ω=0.30)	1.34	1.00	0.31	-1.39	-1.18	0.23	-0.45	0.89	-0.08	0.13
ωDOD$_{60}$-PBEP86-D3BJ (ω=0.22)	1.16	-0.04	-0.14	-1.65	-2.55	0.01	-0.41	0.15	-0.52	-0.51
ωDOD$_{60}$-PBEP86-D3BJ (ω=0.30)	1.97	1.01	0.95	-1.08	-1.65	0.49	-0.25	0.97	0.02	0.31
ωDOD$_{69}$-PBEP86-D3BJ (ω=0.10)	1.10	-1.07	-0.63	-1.85	-4.24	-0.14	-0.32	-0.66	-1.00	-1.14
ωDOD$_{69}$-PBEP86-D3BJ (ω=0.16)	1.49	-0.42	0.10	-1.51	-3.38	0.12	-0.25	-0.08	-0.64	-0.62
ωDOD$_{69}$-PBEP86-D3BJ (ω=0.20)	1.81	-0.03	0.56	-1.30	-2.96	0.29	-0.20	0.26	-0.42	-0.30
ωDOD$_{69}$-PBEP86-D3BJ (ω=0.25)	2.28	0.65	1.27	-0.98	-2.27	0.55	-0.14	0.79	-0.07	0.22
ωDOD$_{69}$-PBEP86-D3BJ (ω=0.30)	2.77	1.15	1.87	-0.66	-1.82	0.82	-0.04	1.20	0.20	0.65
ωDOD$_{72}$-PBEP86-D3BJ (ω=0.08)	1.41	-0.98	-0.26	-1.66	-4.31	0.02	-0.23	-0.54	-0.91	-0.99
ωDOD$_{72}$-PBEP86-D3BJ (ω=0.13)	1.64	-0.61	0.17	-1.48	-3.78	0.16	-0.20	-0.19	-0.71	-0.69
ωnoDispSD$_{20}$-PBEP86 (ω=0.30)	-1.31	-4.91	-4.55	-5.53	-6.40	-1.66	-4.11	-0.90	-2.57	-4.21
ωnoDispSD$_{40}$-PBEP86 (ω=0.30)	-0.28	-3.70	-2.87	-4.10	-5.73	-0.96	-2.96	-0.25	-1.87	-3.07
ωnoDispSD$_{50}$-PBEP86 (ω=0.30)	0.36	-3.10	-1.84	-3.33	-5.35	-0.57	-2.38	0.10	-1.50	-2.45
ωnoDispSD$_{60}$-PBEP86 (ω=0.22)	0.21	-4.13	-2.35	-3.57	-6.79	-0.83	-2.24	-0.75	-1.96	-3.09
ωnoDispSD$_{60}$-PBEP86 (ω=0.30)	1.09	-2.88	-0.92	-2.69	-5.47	-0.24	-1.88	0.23	-1.28	-2.05
ωnoDispSD$_{69}$-PBEP86 (ω=0.10)	0.28	-4.60	-2.47	-3.45	-7.81	-0.86	-1.86	-1.37	-2.20	-3.34
ωnoDispSD$_{69}$-PBEP86 (ω=0.16)	0.65	-4.10	-1.75	-3.10	-7.05	-0.62	-1.78	-0.84	-1.88	-2.88
ωnoDispSD$_{69}$-PBEP86 (ω=0.20)	1.00	-3.65	-1.20	-2.81	-6.55	-0.42	-1.70	-0.46	-1.64	-2.50
ωnoDispSD$_{69}$-PBEP86 (ω=0.25)	1.50	-2.84	-0.31	-2.28	-5.57	-0.06	-1.49	0.16	-1.21	-1.84
ωnoDispSD$_{69}$-PBEP86 (ω=0.30)	1.96	-2.45	0.30	-1.93	-5.18	0.18	-1.35	0.51	-0.96	-1.45
ωnoDispSD$_{72}$-PBEP86 (ω=0.08)	0.60	-4.51	-2.07	-3.20	-7.87	-0.72	-1.68	-1.31	-2.12	-3.18
ωnoDispSD$_{72}$-PBEP86 (ω=0.13)	0.85	-4.11	-1.55	-2.92	-7.23	-0.53	-1.61	-0.91	-1.88	-2.82
PBE10	-3.04	-5.01	-6.64	-6.07	-6.54	-1.66	-4.01	-1.70	-3.64	-4.84
PBE0	-1.12	-1.15	-2.07	-2.72	-1.49	0.11	-2.24	1.72	-1.54	-1.43
PBE32	-0.27	0.69	0.04	-1.24	0.88	0.90	-1.50	3.18	-0.55	0.13
PBE38	0.33	2.11	1.65	-0.12	2.76	1.49	-0.91	4.27	0.22	1.34
PBE50	1.56	5.31	5.12	2.30	7.05	2.75	0.36	6.54	1.93	3.99
PBE60	2.40	7.85	7.69	4.08	10.47	3.68	1.32	8.18	3.27	6.02
r2SCAN	-3.32	-7.09	-8.37	-7.48	-10.55	-2.65	-3.87	-4.59	-4.50	-6.56
r2SCANh	-2.29	-4.79	-5.64	-5.34	-7.37	-1.64	-2.92	-2.39	-3.28	-4.50
r2SCAN0	-0.92	-1.35	-1.67	-2.33	-2.45	-0.23	-1.58	0.65	-1.46	-1.49
r2SCAN30	-0.50	-0.21	-0.40	-1.38	-0.80	0.21	-1.16	1.60	-0.86	-0.52
r2SCAN32	-0.34	0.24	0.10	-1.01	-0.15	0.38	-0.99	1.97	-0.62	-0.13
r2SCAN35	-0.10	0.92	0.85	-0.46	0.84	0.64	-0.75	2.51	-0.26	0.44
r2SCAN50	0.98	4.28	4.34	2.12	5.74	1.86	0.41	5.07	1.51	3.23
r2SCAN60	1.60	6.50	6.45	3.50	8.96	2.61	1.13	6.62	2.65	4.96
TPSS	-4.35	-7.51	-7.69	-4.87	-4.84	-1.56	-2.98	-4.14	-4.38	-5.55
TPSSh	-3.01	-4.87	-4.73	-2.89	-2.06	-0.46	-1.93	-1.73	-3.01	-3.38
TPSS0	-1.19	-0.93	-0.42	-0.09	2.37	1.08	-0.46	1.60	-0.96	-0.18

TPSS30	-0.63	0.38	0.97	0.76	3.84	1.56	-0.03	2.62	-0.28	0.85
TPSS38	0.16	2.32	3.02	2.03	6.08	2.26	0.63	4.11	0.72	2.37
TPSS45	0.89	4.26	4.97	3.24	8.30	2.93	1.25	5.52	1.72	3.86
TPSS50	1.34	5.54	6.22	4.00	9.78	3.36	1.65	6.42	2.37	4.84
TPSS60	2.18	8.09	8.58	5.44	12.71	4.18	2.40	8.12	3.64	6.72
PBE0-D3BJ	-1.77	-3.39	-4.97	-6.21	-7.85	-3.25	-3.10	-1.75	-2.71	-4.24
PBE38-D3BJ	-0.30	-0.01	-1.18	-3.49	-3.42	-1.78	-1.73	0.93	-0.90	-1.36
r2SCAN-D3BJ	-3.59	-8.03	-9.85	-9.17	-13.97	-4.25	-4.31	-6.34	-5.01	-7.90
r2SCANh-D3BJ	-2.58	-5.77	-7.22	-7.11	-10.95	-3.34	-3.38	-4.22	-3.82	-5.91
r2SCAN0-D3BJ	-1.23	-2.41	-3.34	-4.22	-6.23	-2.05	-2.07	-1.29	-2.04	-3.00
r2SCAN50-D3BJ	0.64	3.13	2.55	0.08	1.70	-0.11	-0.11	2.99	0.88	1.60
TPSS-D3BJ	-5.26	-10.59	-11.50	-9.54	-13.10	-6.04	-4.10	-8.74	-5.99	-9.30
TPSSh-D3BJ	-3.88	-7.82	-8.48	-7.44	-10.20	-4.86	-3.02	-6.21	-4.56	-7.03
TPSS0-D3BJ	-2.07	-3.92	-4.05	-4.49	-5.32	-3.18	-1.53	-2.77	-2.51	-3.75
B97-1	-2.31	-2.27	-3.97	-3.16	-1.27	0.29	-1.26	-0.10	-2.09	-2.22
B97-D3BJ	-5.53	-11.12	-10.94	-9.78	-14.14	-5.48	-3.46	-7.75	-6.08	-9.50
B97	-3.95	-5.84	-5.11	-2.39	-1.78	1.47	-1.57	-0.34	-3.34	-3.50
B97-D4	-5.12	-9.59	-9.44	-7.37	-9.84	-3.55	-3.05	-5.59	-5.44	-7.69
PBE-D4	-5.29	-10.17	-12.95	-12.18	-16.10	-6.56	-6.24	-8.03	-6.44	-10.21
revPBE	-3.87	-5.92	-5.70	-3.80	-3.46	0.27	-2.81	-0.26	-3.73	-4.16
revPBE-D3BJ	-5.37	-10.96	-11.36	-10.90	-15.39	-6.46	-4.58	-7.33	-6.34	-9.92
revPBE-D4	-5.70	-11.67	-11.95	-11.23	-15.20	-7.04	-5.19	-8.13	-6.94	-10.40
PBE0-D4	-1.81	-3.45	-5.05	-6.27	-7.72	-3.31	-3.20	-1.87	-2.82	-4.29
B3LYP-D4	-3.04	-5.85	-7.14	-6.03	-7.29	-2.59	-1.67	-4.60	-3.57	-5.32
ωB97X-D3(0)	0.49	2.89	0.21	-2.05	1.00	0.22	-0.16	2.19	0.79	0.79
ωB97M-D4	0.29	2.42	0.52	-1.74	3.57	-0.66	-0.09	1.82	0.43	0.85
PWPB95	-1.01	-1.55	-1.29	-1.36	-1.22	-0.58	-0.16	0.95	-1.09	-1.18
PWPB95-D3BJ	-1.33	-2.63	-2.87	-3.11	-4.51	-2.39	-0.57	-0.83	-1.66	-2.60
PWPB95-D4	-1.42	-2.87	-2.99	-3.37	-4.67	-2.35	-0.82	-1.28	-1.91	-2.80
xDSD$_{75}$-PBEP86-D4	1.49	-1.64	0.05	-1.32	-4.49	0.30	-0.24	-0.55	-1.03	-1.09
xDOD$_{75}$-PBEP86-D4	1.76	-0.69	0.46	-0.99	-3.72	0.54	0.04	-0.33	-0.76	-0.54
ωDOD$_{69}$-PBEP86-D4 (ω=0.10)	1.09	-1.15	-0.75	-1.87	-4.21	-0.10	-0.42	-0.84	-1.12	-1.19
ωDOD$_{60}$-PBEP86-D4 (ω=0.22)	1.09	-0.29	-0.40	-1.70	-2.57	-0.04	-0.54	-0.23	-0.76	-0.66
HF-PBE	4.25	-6.73	7.69	4.49	-6.85	2.84	-2.60	4.86	-2.85	-0.49
HF-PBE-D4	3.29	-9.86	3.99	-0.04	-14.54	-1.41	-3.91	0.22	-4.60	-4.16
HF-PBE0	4.29	-0.65	9.64	5.85	0.70	3.78	-0.81	6.85	-0.13	2.92
HF-PBE0-D4	3.48	-3.31	6.37	1.88	-6.14	0.03	-1.92	2.82	-1.62	-0.29

Table S5: Mean signed deviations (MSDs, kcal/mol) for different DFT functionals on the complete BH9 reaction energy set and its nine subsets. The subsets (or the reaction types) of the BH9 are radical rearrangement (I), Diels-Alder (II), halogen atom transfer (III), hydrogen atom transfer (IV), hydride transfer (V), Boron and Silicon containing reactions (VI), proton transfer (VII), nucleophilic substitution (VIII), and nucleophilic addition (IX). A negative (red) value means underestimation, and a positive (blue) value represents the overestimation of reaction energies.

	MSD (kcal/mol)									
**Functionals**	**I**	**II**	**III**	**IV**	**V**	**VI**	**VII**	**VIII**	**IX**	**Total**
B97M-V	0.16	3.92	-1.99	-0.95	-1.97	-0.31	0.05	0.19	-0.30	0.64
BMK-D3BJ	-2.53	-2.51	-0.52	-0.68	-1.86	-4.00	0.13	0.40	-1.77	-1.81
BMK	-1.26	2.05	-0.87	-0.67	-2.14	1.29	0.20	0.35	1.66	0.30
ωB97X-D	-0.49	0.42	-1.51	-0.51	-0.03	-1.62	0.29	0.25	0.17	-0.27
ωB97X-V	-2.41	-3.52	-0.88	-0.35	0.12	-2.08	0.30	0.58	-2.15	-1.76
ωB97M-V	-0.85	-1.10	-0.93	-0.56	-0.82	-1.40	-0.13	0.50	-0.80	-0.85
ωB97X-2-D3BJ	-0.16	0.59	0.98	-0.07	-0.19	-0.61	0.23	-0.13	-0.46	0.16

ωB97X-2	-0.16	0.59	0.98	-0.07	-0.19	-0.60	0.23	-0.13	-0.46	0.16
ωB97M(2)	-1.18	-1.40	-0.40	-0.34	-0.25	-1.21	0.14	0.19	-1.14	-0.84
PBE-D3BJ	-0.14	3.87	-2.90	-1.04	1.80	-0.45	0.23	-0.42	-1.57	0.74
PBE	0.99	7.73	-3.20	-1.05	1.60	3.60	0.29	-0.40	1.35	2.50
PBE20	-0.94	3.31	-2.48	-0.58	-0.17	2.33	0.06	-0.14	0.16	0.75
LRC-ωPBEh	-2.59	-0.60	-1.94	-0.32	0.50	1.62	0.08	0.19	-0.43	-0.56
PBE0-2-D3BJ	-2.99	-4.54	1.69	0.27	-0.38	-2.20	-0.01	-0.08	-3.45	-1.93
PBE0-2	-2.82	-3.82	1.64	0.28	-0.44	-1.19	-0.01	-0.09	-2.90	-1.59
SOS0-PBE0-2-D3BJ	-1.75	-3.39	1.55	0.26	-0.11	-1.34	0.29	0.18	-2.05	-1.26
SOS0-PBE0-2	-1.12	-1.27	1.38	0.25	-0.21	0.80	0.31	0.22	-0.42	-0.30
PBE0-DH-D3BJ	-3.91	-4.73	-1.10	-0.19	-0.58	-3.14	-0.06	0.18	-4.25	-2.58
PBE0-DH	-2.96	-1.61	-1.33	-0.19	-0.73	0.12	-0.01	0.11	-1.88	-1.15
SOS0-PBE0-DH-D3BJ	-3.26	-3.90	-1.13	-0.22	-0.52	-2.94	0.02	0.25	-3.39	-2.18
SOS0-PBE0-DH	-2.42	-0.84	-1.36	-0.21	-0.70	0.76	0.07	0.18	-1.08	-0.75
PBE-QIDH-D3BJ	-3.56	-4.43	0.22	0.05	-0.56	-2.24	-0.01	0.06	-3.49	-2.16
PBE-QIDH	-3.34	-3.50	0.15	0.05	-0.62	-0.99	0.00	0.04	-2.78	-1.72
SOS1-PBE-QIDH-D3BJ	-2.65	-3.67	0.17	0.01	-0.37	-1.86	0.18	0.29	-2.49	-1.72
SOS1-PBE-QIDH	-2.10	-1.68	0.02	0.02	-0.49	0.45	0.21	0.25	-0.98	-0.80
RSX-QIDH-D3BJ	-6.11	-9.50	0.39	0.17	-0.63	-3.33	-0.08	0.22	-6.19	-4.22
RSX-QIDH	-6.08	-9.38	0.39	0.17	-0.64	-3.14	-0.08	0.22	-6.09	-4.16
RSX-0DH-D3(BJ)	-7.55	-11.46	-0.75	0.06	-0.67	-3.49	-0.09	0.53	-7.06	-5.17
RSX-0DH	-7.52	-11.34	-0.76	0.06	-0.67	-3.30	-0.09	0.53	-6.96	-5.11
BLYP-D3BJ	5.54	11.73	-2.43	-1.26	0.47	1.67	0.53	0.12	3.99	4.19
BLYP	7.85	19.34	-3.01	-1.27	0.08	9.41	0.65	0.14	9.76	7.65
B3LYP-D3BJ	3.26	7.05	-1.81	-0.81	-1.27	0.45	0.29	0.37	2.35	2.28
B3LYP	5.15	13.33	-2.30	-0.82	-1.60	6.92	0.39	0.37	7.11	5.14
BH&HLYP-D3BJ	1.29	2.32	-0.77	-0.33	-2.71	-0.81	0.16	1.08	0.68	0.48
BH&HLYP	2.74	7.16	-1.14	-0.34	-2.96	4.25	0.23	1.04	4.35	2.70
CAM-B3LYP-D3BJ	1.25	2.80	-1.28	-0.56	-1.32	-0.07	0.31	0.66	1.00	0.73
CAM-B3LYP	2.15	6.20	-1.54	-0.55	-1.53	3.91	0.35	0.66	3.56	2.30
B2PLYP-D3BJ	1.72	4.47	-0.53	-0.40	-0.99	0.12	0.32	0.11	1.48	1.46
B2PLYP	2.60	7.49	-0.76	-0.40	-1.16	3.38	0.36	0.09	3.76	2.84
B2GP-PLYP-D3BJ	0.81	2.52	0.29	-0.19	-1.13	-0.24	0.24	0.14	0.60	0.78
B2GP-PLYP	1.45	4.68	0.13	-0.19	-1.24	2.13	0.28	0.09	2.24	1.78
ωB2PLYP-D3BJ	-2.09	-3.05	0.01	-0.16	-1.28	-1.39	0.21	0.45	-2.63	-1.57
ωB2PLYP	-2.07	-2.99	0.01	-0.16	-1.28	-1.27	0.21	0.45	-2.58	-1.53
ωB2G-PLYP-D3BJ	-1.97	-3.00	0.60	-0.05	-1.32	-1.41	0.16	0.34	-2.61	-1.47
ωB2GP-PLYP	-1.97	-2.99	0.60	-0.05	-1.32	-1.41	0.16	0.34	-2.61	-1.47
DSD-PBEP86-D3BJ	-0.54	-0.39	0.90	0.03	-0.28	-0.98	0.22	-0.14	-1.20	-0.26
revDSD-PBEP86-D3BJ	0.11	0.63	0.87	0.02	-0.18	-0.18	0.32	-0.08	-0.27	0.25
revDOD-PBEP86-D3BJ	0.13	0.50	0.83	0.01	-0.14	-0.22	0.36	-0.03	-0.26	0.21
noDispSD-PBEP86	-0.25	0.93	0.94	-0.06	0.00	0.24	0.24	-0.32	-0.17	0.35
$xDSD_{75}$-PBEP86-D3BJ	-0.01	0.54	0.86	-0.06	-0.01	-0.20	0.33	-0.07	-0.17	0.22
$xDOD_{75}$-PBEP86-D3BJ	-0.01	0.26	0.84	-0.08	0.08	-0.30	0.40	-0.01	-0.23	0.12
xnoDispSD-PBEP86	-0.25	0.93	0.94	-0.06	0.00	0.24	0.24	-0.32	-0.17	0.35
ωDSD_{20}-PBEP86-D3BJ (ω=0.30)	-0.51	-0.17	-0.92	-0.27	0.94	-0.81	0.48	0.44	-0.10	-0.20
ωDSD_{40}-PBEP86-D3BJ (ω=0.30)	-0.86	-0.81	-0.36	-0.19	0.75	-0.91	0.49	0.30	-0.45	-0.42
ωDSD_{50}-PBEP86-D3BJ (ω=0.30)	-1.00	-1.13	0.04	-0.13	0.64	-0.89	0.47	0.23	-0.62	-0.51
ωDSD_{60}-PBEP86-D3BJ (ω=0.22)	-0.57	-0.37	0.17	-0.14	0.42	-0.64	0.41	0.14	-0.40	-0.21
ωDSD_{60}-PBEP86-D3BJ (ω=0.30)	-1.09	-1.47	0.57	-0.07	0.53	-0.86	0.45	0.17	-0.79	-0.58
ωDSD_{69}-PBEP86-D3BJ (ω=0.10)	-0.10	0.55	0.37	-0.14	0.07	-0.32	0.37	0.02	-0.16	0.15
ωDSD_{69}-PBEP86-D3BJ (ω=0.16)	-0.35	-0.02	0.56	-0.10	0.25	-0.42	0.38	0.04	-0.31	-0.03
ωDSD_{69}-PBEP86-D3BJ (ω=0.20)	-0.57	-0.52	0.71	-0.07	0.33	-0.53	0.39	0.06	-0.47	-0.20
ωDSD_{69}-PBEP86-D3BJ (ω=0.25)	-0.86	-1.20	0.92	-0.03	0.39	-0.70	0.40	0.09	-0.73	-0.43
ωDSD_{69}-PBEP86-D3BJ (ω=0.30)	-1.08	-1.70	1.17	0.01	0.46	-0.78	0.42	0.09	-0.92	-0.59
ωDSD_{72}-PBEP86-D3BJ (ω=0.08)	-0.09	0.50	0.61	-0.10	0.05	-0.27	0.36	-0.02	-0.17	0.16
ωDSD_{72}-PBEP86-D3BJ (ω=0.13)	-0.29	0.06	0.71	-0.08	0.16	-0.36	0.37	0.01	-0.31	0.02
ωDOD_{20}-PBEP86-D3BJ (ω=0.30)	-0.53	-0.38	-0.94	-0.31	1.02	-0.86	0.52	0.44	-0.14	-0.28

ωDOD$_{40}$-PBEP86-D3BJ (ω=0.30)	-0.89	-1.15	-0.39	-0.23	0.84	-1.01	0.55	0.35	-0.52	-0.54
ωDOD$_{50}$-PBEP86-D3BJ (ω=0.30)	-0.96	-1.41	0.02	-0.17	0.74	-0.97	0.55	0.30	-0.63	-0.60
ωDOD$_{60}$-PBEP86-D3BJ (ω=0.22)	-0.54	-0.59	0.15	-0.17	0.48	-0.70	0.47	0.20	-0.40	-0.27
ωDOD$_{60}$-PBEP86-D3BJ (ω=0.30)	-1.02	-1.77	0.56	-0.10	0.69	-0.98	0.55	0.24	-0.81	-0.66
ωDOD$_{69}$-PBEP86-D3BJ (ω=0.10)	-0.07	0.37	0.36	-0.15	0.12	-0.39	0.42	0.07	-0.16	0.10
ωDOD$_{69}$-PBEP86-D3BJ (ω=0.16)	-0.38	-0.36	0.52	-0.12	0.30	-0.53	0.44	0.12	-0.38	-0.15
ωDOD$_{69}$-PBEP86-D3BJ (ω=0.20)	-0.54	-0.82	0.69	-0.09	0.43	-0.64	0.47	0.13	-0.50	-0.29
ωDOD$_{69}$-PBEP86-D3BJ (ω=0.25)	-0.91	-1.66	0.89	-0.06	0.53	-0.86	0.49	0.16	-0.86	-0.59
ωDOD$_{69}$-PBEP86-D3BJ (ω=0.30)	-1.10	-2.19	1.14	-0.02	0.62	-0.96	0.53	0.18	-1.04	-0.75
ωDOD$_{72}$-PBEP86-D3BJ (ω=0.08)	-0.10	0.22	0.58	-0.12	0.10	-0.38	0.41	0.05	-0.23	0.07
ωDOD$_{72}$-PBEP86-D3BJ (ω=0.13)	-0.28	-0.22	0.68	-0.10	0.24	-0.46	0.43	0.08	-0.34	-0.07
ωnoDispSD$_{20}$-PBEP86 (ω=0.30)	-1.56	0.26	-1.06	-0.27	1.34	0.23	0.39	-0.14	-0.46	-0.12
ωnoDispSD$_{40}$-PBEP86 (ω=0.30)	-1.59	-0.47	-0.39	-0.18	0.93	-0.05	0.38	-0.13	-0.63	-0.34
ωnoDispSD$_{50}$-PBEP86 (ω=0.30)	-1.58	-0.86	0.06	-0.12	0.74	-0.19	0.37	-0.12	-0.75	-0.44
ωnoDispSD$_{60}$-PBEP86 (ω=0.22)	-0.99	0.14	0.22	-0.14	0.52	0.08	0.31	-0.22	-0.40	-0.04
ωnoDispSD$_{60}$-PBEP86 (ω=0.30)	-1.36	-0.98	0.67	-0.06	0.65	-0.20	0.37	-0.15	-0.71	-0.39
ωnoDispSD$_{69}$-PBEP86 (ω=0.10)	-0.49	0.95	0.42	-0.13	0.09	0.23	0.25	-0.28	-0.22	0.27
ωnoDispSD$_{69}$-PBEP86 (ω=0.16)	-0.71	0.35	0.61	-0.09	0.26	0.13	0.27	-0.24	-0.34	0.09
ωnoDispSD$_{69}$-PBEP86 (ω=0.20)	-0.85	-0.10	0.79	-0.06	0.39	0.04	0.30	-0.22	-0.43	-0.04
ωnoDispSD$_{69}$-PBEP86 (ω=0.25)	-1.07	-0.76	1.01	-0.02	0.46	-0.11	0.32	-0.16	-0.63	-0.26
ωnoDispSD$_{69}$-PBEP86 (ω=0.30)	-1.18	-1.20	1.28	0.01	0.53	-0.22	0.35	-0.14	-0.75	-0.38
ωnoDispSD$_{72}$-PBEP86 (ω=0.08)	-0.40	0.89	0.68	-0.09	0.06	0.22	0.25	-0.29	-0.21	0.29
ωnoDispSD$_{72}$-PBEP86 (ω=0.13)	-0.54	0.52	0.78	-0.07	0.19	0.19	0.27	-0.26	-0.25	0.18
PBE10	0.01	5.49	-2.83	-0.79	0.49	2.98	0.16	-0.30	0.81	1.60
PBE0	-1.39	2.28	-2.32	-0.49	-0.44	2.01	0.01	-0.03	-0.16	0.35
PBE32	-2.09	0.70	-2.06	-0.37	-0.75	1.50	-0.04	0.09	-0.75	-0.27
PBE38	-2.62	-0.50	-1.88	-0.28	-0.98	1.10	-0.07	0.21	-1.22	-0.74
PBE50	-3.83	-3.22	-1.52	-0.10	-1.47	0.15	-0.13	0.50	-2.35	-1.83
PBE60	-4.82	-5.39	-1.30	0.02	-1.83	-0.66	-0.15	0.76	-3.33	-2.71
r2SCAN	0.09	4.34	-2.34	-0.77	0.07	0.42	0.26	-0.17	-0.33	1.01
r2SCANh	-0.59	2.86	-2.13	-0.59	-0.66	0.18	0.16	-0.04	-0.61	0.43
r2SCAN0	-1.60	0.66	-1.83	-0.37	-1.31	-0.23	0.02	0.17	-1.19	-0.42
r2SCAN30	-1.93	-0.07	-1.73	-0.31	-1.48	-0.38	-0.02	0.25	-1.42	-0.70
r2SCAN32	-2.07	-0.36	-1.70	-0.28	-1.55	-0.44	-0.04	0.29	-1.51	-0.81
r2SCAN35	-2.27	-0.80	-1.65	-0.25	-1.64	-0.53	-0.06	0.34	-1.66	-0.98
r2SCAN50	-3.29	-2.97	-1.44	-0.09	-2.05	-1.01	-0.16	0.60	-2.47	-1.83
r2SCAN60	-3.98	-4.40	-1.35	0.01	-2.28	-1.36	-0.21	0.79	-3.06	-2.40
TPSS	2.39	11.21	-2.82	-0.91	1.31	5.94	0.45	-0.46	4.41	4.14
TPSSh	1.36	8.79	-2.50	-0.69	0.39	5.11	0.32	-0.30	3.62	3.15
TPSS0	-0.20	5.14	-2.03	-0.43	-0.43	3.79	0.16	-0.02	2.21	1.69
TPSS30	-0.70	3.93	-1.86	-0.35	-0.63	3.33	0.12	0.07	1.69	1.21
TPSS38	-1.50	2.10	-1.64	-0.24	-0.95	2.62	0.06	0.24	0.88	0.47
TPSS45	-2.30	0.27	-1.46	-0.14	-1.25	1.89	0.01	0.43	0.04	-0.28
TPSS50	-2.84	-0.95	-1.35	-0.08	-1.44	1.40	-0.01	0.55	-0.54	-0.78
TPSS60	-3.93	-3.39	-1.18	0.03	-1.82	0.38	-0.05	0.82	-1.74	-1.80
PBE0-D3BJ	-2.38	-1.27	-2.05	-0.49	-0.23	-1.91	-0.04	-0.04	-2.85	-1.28
PBE38-D3BJ	-3.52	-3.83	-1.63	-0.29	-0.78	-2.68	-0.11	0.21	-3.72	-2.28
r2SCAN-D3BJ	-0.26	2.89	-2.23	-0.77	0.16	-1.38	0.25	-0.16	-1.43	0.33
r2SCANh-D3BJ	-0.95	1.35	-2.02	-0.60	-0.56	-1.73	0.14	-0.03	-1.76	-0.28
r2SCAN0-D3BJ	-1.99	-0.98	-1.71	-0.38	-1.20	-2.28	0.00	0.19	-2.43	-1.18
r2SCAN50-D3BJ	-3.72	-4.74	-1.31	-0.09	-1.93	-3.23	-0.18	0.61	-3.82	-2.66
TPSS-D3BJ	0.96	6.26	-2.43	-0.91	1.59	0.66	0.37	-0.48	0.67	1.87
TPSSh-D3BJ	0.04	4.09	-2.13	-0.69	0.67	-0.03	0.25	-0.32	0.07	0.99
TPSS0-D3BJ	-1.64	0.28	-1.65	-0.42	-0.18	-1.29	0.09	-0.02	-1.48	-0.53
B97-1	2.02	7.31	-2.21	-0.79	-1.10	2.98	0.18	-0.03	2.71	2.41
B97-D3BJ	4.17	9.70	-2.64	-1.01	1.21	1.31	0.43	-0.20	2.81	3.40
B97	7.09	18.77	-3.33	-1.06	0.83	9.75	0.56	-0.07	9.80	7.49
B97-D4	4.66	11.80	-2.95	-0.99	0.70	3.87	0.44	-0.08	4.55	4.34

PBE-D4	-0.37	3.37	-2.94	-1.02	1.56	-0.53	0.22	-0.45	-1.92	0.51
revPBE	3.42	13.11	-3.32	-0.98	1.94	7.64	0.45	-0.24	6.29	5.08
revPBE-D3BJ	0.72	4.59	-2.67	-0.94	2.32	-0.50	0.32	-0.34	-0.25	1.22
revPBE-D4	-0.64	1.94	-2.72	-0.85	1.55	-0.83	0.26	-0.45	-2.18	0.05
PBE0-D4	-2.55	-1.60	-2.09	-0.48	-0.45	-1.84	-0.05	-0.08	-3.07	-1.43
B3LYP-D4	3.01	6.60	-1.89	-0.78	-1.64	0.71	0.27	0.30	2.09	2.08
ωB97X-D3(0)	-0.79	0.15	-1.37	-0.42	0.36	-0.96	0.34	0.32	-0.14	-0.29
ωB97M-D4	-2.18	-3.60	-1.03	-0.33	0.04	-2.78	0.30	0.75	-2.34	-1.84
PWPB95	0.12	3.74	-1.09	-0.51	-0.55	2.11	0.12	-0.07	2.00	1.20
PWPB95-D3BJ	-0.31	2.08	-0.97	-0.52	-0.44	0.04	0.10	-0.04	0.74	0.42
PWPB95-D4	-0.78	1.16	-0.96	-0.48	-0.70	0.03	0.08	-0.13	-0.06	0.02
xDSD$_{75}$-PBEP86-D4	0.12	0.79	0.80	-0.04	-0.22	0.13	0.34	-0.05	0.01	0.32
xDOD$_{75}$-PBEP86-D4	0.20	0.70	0.78	-0.06	-0.14	0.14	0.40	-0.01	0.08	0.31
ωDOD$_{69}$-PBEP86-D4 (ω=0.10)	-0.10	0.35	0.35	-0.13	0.00	-0.22	0.42	0.01	-0.22	0.09
ωDOD$_{60}$-PBEP86-D4 (ω=0.22)	-0.60	-0.71	0.15	-0.14	0.32	-0.55	0.48	0.15	-0.56	-0.33
HF-PBE	-0.64	7.65	1.63	2.32	0.02	6.85	-0.66	-1.24	4.08	3.66
HF-PBE-D4	-2.46	2.06	1.97	2.37	-0.11	1.98	-0.74	-1.36	-0.14	1.12
HF-PBE0	-2.74	2.25	0.91	1.75	-0.80	4.06	-0.51	-0.44	1.15	1.13
HF-PBE0-D4	-4.19	-2.39	1.19	1.78	-0.87	-0.21	-0.58	-0.52	-2.34	-0.99

Table S6: Mean absolute deviations (MADs, kcal/mol) of a few additional functionals evaluated on the complete BH9 barrier height set and its nine subsets. The subsets (or the reaction types) of the BH9 are radical rearrangement (I), Diels-Alder (II), halogen atom transfer (III), hydrogen atom transfer (IV), hydride transfer (V), Boron and Silicon containing reactions (VI), proton transfer (VII), nucleophilic substitution (VIII), and nucleophilic addition (IX). A negative (red) value means underestimation, and a positive (blue) value represents the overestimation of reaction energies.

	MAD (kcal/mol)									
**Functionals**	**I**	**II**	**III**	**IV**	**V**	**VI**	**VII**	**VIII**	**IX**	**Total**
B97	4.77	11.15	6.27	4.57	5.24	6.60	3.36	2.92	5.34	6.99
B97-D3BJ	5.54	11.17	10.94	9.78	14.36	6.17	3.49	7.75	6.31	9.61
B97-D4	5.13	9.83	9.44	7.40	10.03	4.98	3.48	5.59	5.73	7.92
PBE	4.52	8.00	9.80	8.50	9.87	3.80	5.48	4.35	5.17	7.41
PBE-D3BJ	5.22	10.01	12.74	12.02	16.20	6.42	6.11	7.80	6.36	10.09
PBE-D4	5.29	10.17	12.95	12.18	16.20	6.64	6.24	8.03	6.50	10.23
revPBE	4.21	9.10	6.85	5.32	5.78	5.27	4.38	3.08	4.59	6.43
revPBE-D3BJ	5.37	10.97	11.37	10.90	15.55	6.55	4.59	7.33	6.44	9.95
revPBE-D4	5.70	11.67	11.95	11.23	15.38	7.14	5.19	8.13	6.94	10.43
PBE0	1.56	2.85	2.89	3.35	2.99	1.86	2.72	1.98	1.81	2.66
PBE0-D3BJ	2.10	3.73	4.97	6.21	7.88	3.27	3.10	1.75	2.87	4.40
PBE0-D4	2.16	3.84	5.05	6.27	7.75	3.33	3.20	1.87	2.96	4.46
B3LYP	2.94	7.47	3.69	3.11	3.74	4.98	1.57	1.91	3.65	4.67
B3LYP-D3BJ	3.09	6.11	7.08	6.04	7.71	3.79	1.58	4.53	3.70	5.54
B3LYP-D4	3.11	6.05	7.14	6.03	7.43	3.69	1.73	4.60	3.76	5.51
ωB97X-D3(0)	1.03	2.94	1.40	2.34	1.66	1.68	0.55	2.51	1.13	2.08
ωB97X-D3BJ	1.07	2.72	1.24	1.89	3.84	2.01	0.58	2.35	1.21	2.14
ωB97X-D4	1.19	2.72	1.03	1.92	3.90	2.01	0.63	2.01	1.35	2.14
PWPB95	1.21	2.56	1.70	1.82	1.96	1.79	1.10	1.45	1.42	1.93
PWPB95-D3BJ	1.47	2.78	2.88	3.12	4.55	2.40	0.92	1.12	1.74	2.70
PWPB95-D4	1.56	2.93	2.99	3.37	4.72	2.36	1.01	1.37	1.95	2.85
$xDSD_{75}$-PBEP86-D3BJ	2.14	2.31	1.18	2.02	5.70	0.91	0.59	0.88	1.30	2.19
$xDSD_{75}$-PBEP86-D4	2.15	1.73	1.08	1.69	4.50	0.81	0.49	0.67	1.07	1.79
$xDOD_{75}$-PBEP86-D3BJ	2.29	1.30	1.15	1.82	4.81	0.76	0.45	0.68	1.00	1.73
$xDOD_{75}$-PBEP86-D4	2.29	1.04	1.21	1.60	3.73	0.81	0.43	0.55	0.82	1.49
ωDOD_{69}-PBEP86-D3BJ (ω=0.10)	1.72	1.25	1.02	1.96	4.26	0.63	0.53	0.73	1.06	1.61
ωDOD_{69}-PBEP86-D4 (ω=0.10)	1.71	1.30	1.09	1.98	4.23	0.64	0.60	0.88	1.16	1.64
ωDOD_{60}-PBEP86-D3BJ (ω=0.22)	1.61	0.80	0.83	1.80	2.57	0.61	0.57	0.78	0.69	1.23
ωDOD_{60}-PBEP86-D4(ω=0.22)	1.57	0.87	0.91	1.82	2.59	0.62	0.66	0.80	0.85	1.27

Table S7: Mean absolute deviations (MAD, in kcal/mol) of a few additional functionals evaluated on the complete BH9 reaction energy set and its nine subsets. The subsets (or the reaction types) of the BH9 are radical rearrangement (I), Diels-Alder (II), halogen atom transfer (III), hydrogen atom transfer (IV), hydride transfer (V), Boron and Silicon containing reactions (VI), proton transfer (VII), nucleophilic substitution (VIII), and nucleophilic addition (IX). A negative (red) value means underestimation, and a positive (blue) value represents the overestimation of reaction energies.

	MAD (kcal/mol)									
**Functionals**	**I**	**II**	**III**	**IV**	**V**	**VI**	**VII**	**VIII**	**IX**	**Total**
B97	7.18	19.98	6.19	2.87	2.19	10.74	2.83	2.63	9.80	9.92
B97-D3BJ	4.30	11.21	5.13	2.55	2.47	2.16	2.83	2.19	3.49	5.69
B97-D4	4.75	13.12	5.48	2.66	2.14	4.58	2.74	2.46	4.69	6.63
PBE	2.68	8.89	6.03	3.02	2.64	4.41	2.85	2.57	2.99	5.15
PBE-D3BJ	1.89	5.35	5.63	2.91	2.81	1.39	2.87	2.74	3.61	3.73
PBE-D4	1.86	4.93	5.65	2.92	2.63	1.35	2.81	2.84	3.71	3.59
revPBE	4.26	14.00	6.43	3.14	2.94	8.65	2.89	3.14	6.29	7.55
revPBE-D3BJ	1.80	6.08	5.44	2.87	3.32	1.76	2.89	2.95	2.89	3.96
revPBE-D4	1.59	3.88	5.39	2.85	2.71	1.57	2.69	3.09	3.68	3.22
PBE0	2.16	4.04	3.52	1.95	1.47	2.70	1.63	1.44	1.75	2.75
PBE0-D3BJ	2.43	2.35	3.10	1.84	1.25	2.20	1.65	1.46	3.44	2.23
PBE0-D4	2.59	2.49	3.14	1.85	1.33	2.13	1.60	1.50	3.61	2.31
B3LYP	5.15	14.27	3.85	2.06	2.34	7.66	1.68	1.59	7.11	7.10
B3LYP-D3BJ	3.33	8.18	3.11	1.83	2.11	1.61	1.69	1.80	2.61	4.14
B3LYP-D4	3.08	7.70	3.16	1.84	2.35	1.60	1.61	1.67	2.44	3.98
ωB97X-D3(0)	1.50	1.57	2.06	1.67	0.90	1.95	1.07	1.22	1.17	1.55
ωB97X-D3BJ	1.69	2.82	1.54	1.84	0.90	3.20	1.01	1.68	1.99	2.10
ωB97X-D4	2.18	3.80	1.45	1.76	0.83	3.37	0.95	1.51	2.45	2.46
PWPB95	1.36	4.18	2.09	1.45	1.13	2.52	1.04	1.05	2.00	2.42
PWPB95-D3BJ	1.07	2.57	1.90	1.40	1.04	1.04	1.05	0.90	1.17	1.68
PWPB95-D4	1.13	1.82	1.87	1.37	1.19	0.95	1.01	0.77	1.10	1.44
xDSD$_{75}$-PBEP86-D3BJ	2.19	0.90	1.85	1.84	0.59	0.86	0.52	0.53	0.79	1.26
xDSD$_{75}$-PBEP86-D4	2.02	1.03	1.77	1.74	0.54	0.66	0.48	0.48	0.75	1.23
xDOD$_{75}$-PBEP86-D3BJ	2.09	0.70	1.85	1.80	0.59	0.82	0.55	0.52	0.77	1.17
xDOD$_{75}$-PBEP86-D4	1.92	0.92	1.79	1.74	0.55	0.63	0.57	0.48	0.71	1.18
ωDOD$_{69}$-PBEP86-D3BJ (ω=0.10)	1.59	0.78	1.38	1.48	0.60	0.80	0.63	0.56	0.78	1.04
ωDOD$_{69}$-PBEP86-D4 (ω=0.10)	1.62	0.77	1.40	1.49	0.55	0.66	0.61	0.54	0.80	1.03
ωDOD$_{60}$-PBEP86-D3BJ (ω=0.22)	1.56	1.12	1.19	1.28	0.74	1.00	0.71	0.79	0.76	1.12
ωDOD$_{60}$-PBEP86-D4(ω=0.22)	1.60	1.20	1.23	1.28	0.61	0.85	0.69	0.71	0.88	1.13
